# First Report and Comparative Genomics Analysis of a *bla*_OXA-244_-Harboring *Escherichia coli* Isolate Recovered in the American Continent

**DOI:** 10.3390/antibiotics8040222

**Published:** 2019-11-13

**Authors:** Deisy Abril, Ingrid Gisell Bustos Moya, Ricaurte Alejandro Marquez-Ortiz, Diego Fernando Josa Montero, Zayda Lorena Corredor Rozo, Isabel Torres Molina, Natasha Vanegas Gómez, Javier Escobar-Perez

**Affiliations:** 1 Bacterial Molecular Genetics Laboratory, Universidad El Bosque, Carrera 9 Nº131A-02, Bogota D.C. 110121, Colombia; djabril@unbosque.edu.co (D.A.); ramarquezo@gmail.com (R.A.M.-O.); zcorredor@unbosque.edu.co (Z.L.C.R.); natashavanegas@yahoo.es (N.V.G.); 2 Grupo de Medicina Cardiovascular y especialidades de alta complejidad—Fundación Clínica Shaio, Bogota D.C. 110121, Colombia; ingrid.bustos@shaio.org (I.G.B.M.); diego.josa@shaio.org (D.F.J.M.); isabel.torres@shaio.org (I.T.M.); 3 The i3 institute, Faculty of Science University of Technology, Sydney PO Box 123, Australia

**Keywords:** *bla_OXA-244_*, *Escherichia coli*, carbapenems, resistance, Colombia

## Abstract

The carbapenemase OXA-244 is a derivate of OXA-48, and its detection is very difficult in laboratories. Here, we report the identification and genomic analysis of an *Escherichia coli* isolate (28Eco12) harboring the *bla*_OXA-244_ gene identified in Colombia, South America. The 28Eco12 isolate was identified during a retrospective study, and it was recovered from a patient treated in Colombia. The complete nucleotide sequence was established using the PacBio platform. A comparative genomics analysis with other *bla*_OXA-244_–harboring *Escherichia coli* strains was performed. The 28Eco12 isolate belonged to sequence type (ST) 38, and its genome was composed of two molecules, a chromosome of 5,343,367 bp and a plasmid of 92,027 bp, which belonged to the incompatibility group IncY and did not harbor resistance genes. The *bla*_OXA-244_ gene was chromosomally encoded and mobilized by an ISR1-related Tn*6237* composite transposon. Notably, this transposon was inserted and located within a new genomic island. To our knowledge, this is the first report of a *bla*_OXA-244_–harboring *Escherichia coli* isolate in America. Our results suggest that the introduction of the OXA-244-producing *E. coli* isolate was through clonal expansion of the ST38 pandemic clone. Other isolates producing OXA-244 could be circulating silently in America.

## 1. Introduction

The World Health Organization WHO has recognized carbapenem-resistant *Enterobacteriaceae* as pathogens with critical priority for the development of new antibiotics [1]. OXA-244, a carbapenemase belonging to the Class D family, is a derivate of OXA-48 and encoded by the *bla*_OXA-244_ gene. Although there are multiple reports of OXA-48-producing isolates, reports of isolates harboring OXA-244 are less frequent, perhaps because their detection is difficult due to their reduced carbapenem activity. The *bla*_OXA-244_ gene was initially described in 2011, within a *Klebsiella pneumoniae* isolate, which was identified in Spain [2]. It has already been identified in *Escherichia coli* isolates recovered from Germany [3], France [4,5], the United Kingdom [6], Southeast Asia [7], and Egypt [5]. The molecular characterization of some of these *E. coli* isolates have shown that the majority of them belong to sequence type (ST) 38, although recently other STs have been found (ST361, ST1722, and ST3541) [5]; and they contain other β-lactamases, such as TEM, CTX-M, and CMY. The *bla*_OXA-244_ gene is located in the chromosome within a truncated Tn*1999.2* transposon, which is immersed into an IS*R1*-based Tn*6237* transposon [4,8]. Here, we provide a genomic analysis of an *Escherichia coli* isolate (28Eco12) containing the *bla*_OXA-244_ gene that was recovered from a patient in Colombia, South America. To our knowledge, this is the first report of a *bla*_OXA-244_–harboring *Escherichia coli* isolate in America.

## 2. Results

**Figure 1:**
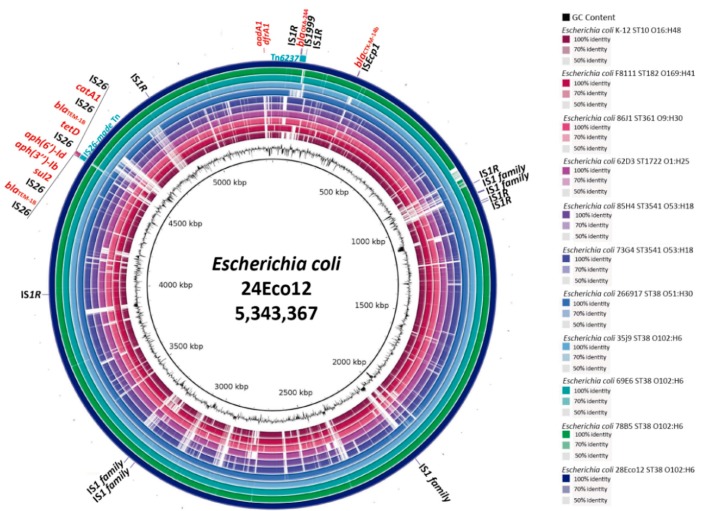
BLASTn comparison of the *bla*OXA-244-containing *Escherichia coli* chromosomes. The K-12 (GenBank accession number NC_000913), F8111-1SC3 (GenBank accession number NZ_CP024269), and 266917_2 (GenBank accession number NZ_CP026723.1) strains were used as references. At the more external circle is shown the localization of the resistance genes and their putative genetic platforms of mobilization. The positions of the seven identical ISR1 and five IS1-family (89% of identity) sequences are also indicated. The strain positions on the figure are as follow (internal to external) (sequence type/serotype): K12 (ST10/O16:H48), F8111-1SC3 (ST182/O169:H41), 86J1 (ST361/O9:H30) MKGU01, 62D3 (ST1722/O1:H25) MKGY01, 85H4 (ST3541/O53:H18) MKGW01, 73G4 (ST3541/O53:H18) MKGV01, 266917_2 (ST38/O51:H30), 35J9 (ST38/O102:H6) MKGX01, 69E6 (ST38/O102:H6) MKGZ01, 78B5 (ST38/O102:H6) MKGT01, and 28Eco12 (ST38/O102:H6) NZ_CP038505.

The 28Eco12 isolate was identified from a retrospective study in Bogotá, Colombia (see Materials and Methods), and we decided to establish its complete genome to determine its resistome and mobile genetic platform distribution (IS content). The genome was composed of two molecules, a chromosome of 5,343,367 bp and a plasmid of 92,027 bp (p28Eco12), which belonged to the incompatibility group IncY and did not harbor resistance genes. The resistance-genes arsenal of the isolate was composed of *aph(3′’)-Ib, aph(6)-Id*, *aaaA1* (aminoglycosides), *bla*_OXA-244_, *bla_CTX-M-14b_*, *bla_TEM-1b_* (beta-lactams), *catA1* (chloramphenicol), *sul2* (sulphonamides), *dfrA1* (trimethoprim)*,* and *tetD* (tetracycline) genes, all chromosomally encoded (Figure 1). The 28Eco12 isolate belonged to ST38 [9]. The in silico serotyping of the isolate was O102:H6.

**Figure 2:**
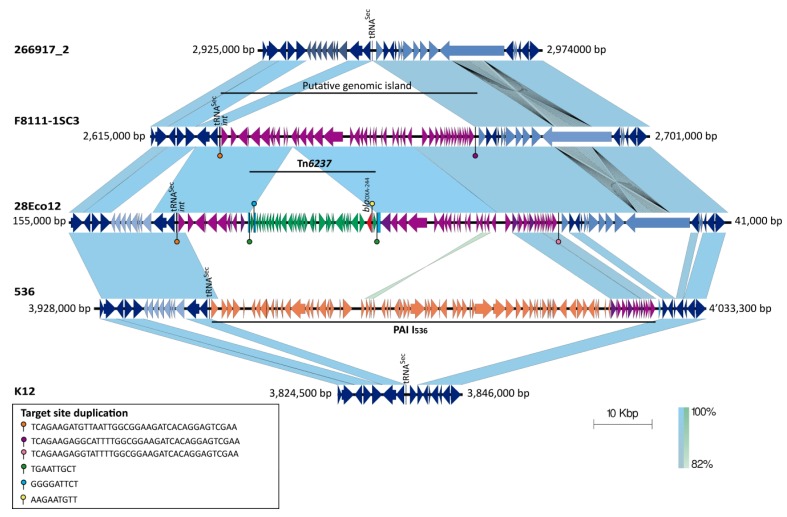
Comparison of the region where the *bla*_OXA-244_ gene was inserted within *Escherichia coli* 28Eco12 isolate. The red arrow corresponds to the *bla*_OXA-244_ gene. The mobile genetics elements are shown in different colors. The putative genomic island is shown in purple and its insertion within the *tRNA-sec* gene is indicated respect to the *E. coli* strain 266917_2 (GenBank accession number CP026723.1), F8111-1SC3 (GenBank accession number NZ_CP024269), 536-EC15 (GenBank accession number HG977710.1), and K-12 (GenBank accession number NC_000913). The blue rectangles correspond to the gene where the Tn*6237* transposon was inserted (green arrows). The pallets represent the target-site duplications. The *int* gene that encodes the phage integrase protein is shown. Blue shading between pairs of sequences indicates >90% of identity in a window of 400 bp. The scale bar indicates sequence length.

Using the complete genome sequence, the 28Eco12 isolate was found to have a close genetic relationship with the *E. coli* strain 266917_2 (ST38), described recently in the United Kingdom (90% coverage, 97% identity, GenBank accession number CP026723.1), which does not contain the *bla*_OXA-244_ gene. The genomic comparative analysis revealed that the *bla*_OXA-244_ gene was mobilized by the Tn*6237* transposon, as it has previously been described in *Escherichia coli* strain VAL [4,8]. However, in the 28Eco12 isolate, the Tn*6237* transposon was not inserted within the II_536_ pathogenicity island, as was previously reported to *bla*_OXA-48_ [8], but into a new putative genomic island, inserted within the *tRNA-sec* gene. Its insertion produced a 39 bp direct repeat sequence (TTCGACTCCTGTGATCTTCCGCCAATTAACATCTTCTGA). This event did not change the *tRNA-sec* gene sequence (Figure 2).

The putative island was also present in the *bla*_OXA-244_-negative enterotoxigenic *E. coli* F8111-1SC3 isolate (GenBank accession number NZ_CP024269). Interestingly, the *tRNA-sec* gene is a hot spot for DNA insertion, because it also serves as the insertion site of the I_536_ pathogenicity island in the uropathogenic strain *E. coli* 536 [10]. These results suggest that the Tn*6237* transposon is active and moves to different sites in the *E. coli* chromosome. In addition, the isolate harbored 69 insertion sequences (IS) belonging to 17 different IS families (Table 1). Some of these present as single copy, partial form, or multiple copies. The most frequent IS families were IS1, IS200/IS605_ssgr_IS200, and IS3, with 13, 10, and 8 IS copies, respectively. Target site duplications (TSD) are signatures of transposition events, and among the 69 ISs, 25 presented TSDs and none were present within the *E. coli* F8111-1SC3 isolate, indicating that they were inserted by single-copy transposition. The TSD pattern analysis also revealed the presence of two composite transposons, the Tn*6237* (mentioned previously) and a 15,730 bp IS26-made transposon, which was inserted within a gene that encodes a hypothetical protein and mobilizes the *aph(3′’)-Ib, aph(6)-Id, bla_TEM-1b_* (two copies), *catA1*, *sul2*, and *tetD* genes. Notably, this IS26 transposon was also inserted within another putative genomic island, which was inserted into the *tRNA-leu* gene. The comparative analysis suggested that this IS26 transposon was mobilized from a plasmid because it harbored the *repA* gene that corresponds to the incompatibility group IncQ-1 and possesses DNA fragments with a high percentage of identity to pD90-1 and pEC141 plasmids, which were identified in *mcr-1*-containing *Salmonella enterica* and *E. coli* strains, respectively [11]. With respect to the other resistance genes, the *bla*_CTX-M_ gene was mobilized by ISEcp1 and an IS26 remnant, which were inserted within a gene that encodes a hypothetical protein.

## 3. Discussion

In this study, we perform the first report of an *Escherichia coli* isolate carrying the *bla*_OXA-244_ gene in Colombia, South America. These *bla*_OXA-244_-positive isolates are less frequent (or perhaps they circulate but are not detected) by their difficult detection and clonal dissemination. The multiresistant 28Eco12 isolate harbored only the phage-like IncY plasmid p28Eco12, which is genetically related to the plasmids p266917_2_02 (88% coverage, 99% identity, GenBank accession number CP026725.1), p1303_95 (91% coverage, 99% identity, GenBank accession number CP009168.1), p1 of *Salmonella enterica* strain ty3-243 (90% coverage, 93% identity, GenBank accession number LT905089.1), and the *bla*_KPC_-containing pCRKP-59-KPC (89% coverage, 94% identity, GenBank accession number KX928752.1). Although this plasmid does not transport resistance genes, it appears to be conserved in almost all *bla*_OXA-244_-containing *E. coli* strains included in our analysis, and its permanence is perhaps caused by the presence of the P1 *phd-doc* toxin-antitoxin system that participates in host post-segregational killing [12]. Currently, there is limited knowledge about this phage-like IncY-plasmid family (for instance, the 37% of their ORFs is encoding for hypothetical proteins), but it is also becoming a genetic platform to transport important resistance genes, such as *bla*_CTX-M-15_ and *mcr-1*; the latter confers resistance to colistin [13].

All resistant genes were chromosomally located and mobilized by active composite transposons, such as Tn*6237*, which has moved to different sites in the *E. coli* chromosome. In *E. coli*, the *bla*_OXA-244_ gene was disseminated mainly by ST38 clone in Europe and Asia [3,4,5,6,7]. However, non-ST38 *E. coli* isolates are starting to appear in other countries, showing some genetic differences (Figure 1). As it is known that ISs have an important impact on genetic variability, genome structure and function, and foreign DNA acquisition, we try to decipher the potential of the 28Eco12 isolate to capture and move more resistance genes through an analysis of the IS content and their TSD and flanking-sequences patterns. Notably, this isolate has incorporated at least 69 ISs, showing a IS massive expansion process [14]; the ISs-belonging family IS1 was the most active, with fifteen copies, in which four copies probably were recently mobilized as single transposition events (unique copies) and two mobilized as a composite transposon and responsible of the *bla*_OXA-244_-gene integration (Table 1). In spite of finding five IS26 copies, only two of these were mobilized as a composite transposon and transported seven resistance genes. A study conducted by He et al. reported the IS26 participation in the plasmid reorganization from clinical strains [15]. The high IS content found in this multiresistant *E. coli* isolate indicates a high likelihood to acquire more resistance genes.

Finally, our institution searched for the presence of the *bla*_OXA-244_ gene within other carbapenem-resistant *E. coli* isolates from 2013 to the present day, but none were positive. Considering the time of the identification of the isolate, we believe that the *E. coli* isolate could have been acquired in the remittent institution, suggesting an inter-institution dissemination. No additional information could be obtained from the other institution.

## 4. Materials and Methods

The 28Eco12 isolate was identified from a retrospective study, conducted to characterize the molecular mechanisms in carbapenem-resistant *Enterobacteriaceae* isolates, which were recovered between 2013 and 2017, from a health institution in Bogotá, Colombia. The 28Eco12 isolate was recovered from a male patient, in September 2013, who was transferred from another health institution in the same city. The patient had suffered multiple traumas caused by a fall from a height of 20 m, and he required treatment in the intensive-care unit for eleven days. The patient was transferred to our institution, however, on the next day; the patient had fever, dysuria, urethral pain, leukocytosis, and urethral purulent secretion, suggesting a possible catheter-associated urinary tract infection. From a urine sample, the carbapenem-resistant *Escherichia coli* isolate 28Eco12 was identified, which was also resistant to ampicillin/sulbactam, cefotaxime, ceftriaxone, cefepime, and aztreonam. The Hodge Test was positive, and synergy and double-disc tests with boronic acid and EDTA were negative. The patient was treated with meropenem (2 g every 8 h) and colistin (100 mg every 8 h), and thirteen days later, he responded well to the treatment. No history of travel by him or his relatives was reported.

The complete genome sequence of the *bla*_OXA-244_-positive 28Eco12 isolate was obtained using the PacBio RS II platform (Pacific Biosciences of California, Inc., Menlo Park, CA, USA) and assembled through the previously reported procedure [16]. Briefly, sequencing reads were de novo assembled, using the HGAP 3 protocol, and manually verified using BWA-MEM (Burrows–Wheeler Aligner with maximal exact matches) [17] and Tablet v1.15.09.01 [18]. Misassembled terminal repeat overlap sequences were identified with Gepard (Genome Pair Rapid Dotter) [19] and trimmed manually. The genome was annotated using Prokka v1.11 [20], and the relevant regions were manually confirmed using BLASTn and BLASTp and edited in Artemis [21]. The resistance-gene arsenal was identified using ARIBA (https://github.com/sanger-pathogens/ariba/wiki), ResFinder [22], CARD [23], and ARG-ANNOT databases [24]. The insertion sequences (IS) were found using ISsaga (http://issaga.biotoul.fr/), and their flanking sequences were manually determined.

The study was approved by the ethics committee of the Shaio Clinic. The 28Eco12 complete genome sequenced in this study is available in the DDBJ/EMBL/GenBank public databases, under the accession numbers CP038505.1 and CP038506.1.

## 5. Conclusions

The isolates producing OXA-244 could be circulating in America and may not yet be identified, perhaps due to their very low frequency, very difficult detection, or weakness in antimicrobial resistance surveillance programs in some countries (such as Colombia). It is necessary to strengthen the surveillance of last-line antibiotic resistance and move toward the implementation of molecular and genomic tools for the detection of resistance genes in clinical settings.

## Figures and Tables

**Table 1:** Insertion sequences identified in 28Eco12 isolate. Target site duplications (TSD) are shown in bold and underlined.

IS Family	IS	Position	Right and Left Flanking Sequences		Comments
IS1	IS1R	102025..102792	**TGAATTGCT**	AAGAATGTT	Composite transposon harboring the *bla*_OXA-244_ gene.
	IS1R	123120..123887	GGGGATTCT	**TGAATTGCT**	
	IS1R	936063..936830	**CAGACAACG**	**CAGACAACG**	Single IS transposition. IS inserted within a putative prophage
	IS1-like	975280..976060	GTCGCAACC	TACAACGTT	IS inserted within a putative prophage
	IS1-like	977300..978080	GACAATGTC	CAATCTGCT	IS inserted within a putative prophage
	IS1R	1007836..1008603	**TGCTTTTCT**	**TGCTTTTCT**	Single IS transposition. IS inserted within an intergenic region
	IS1R	1015519..1016286	**GCCAATTCG**	**GCCAATTCG**	Single IS transposition. IS inserted within the *cmtB* gene
	IS1-like	2087231.. 2087998	CGGTTTTGG	GAAGAGTTC	IS inserted within the *hchA* gene
	IS1-like	3237236..3237910	-	GAAATCCCC	IS (truncated) inserted within a putative prophage
	IS1-like	3266386..3267153	CTGCAAATC	TACAACCGG	IS inserted within a putative prophage
	IS1R	3972674..3973441	**CTGCTCCTG**	**CTGCTCCTG**	Single IS transposition. IS inserted within a hypothetical gene
	IS1R	4845817..4846584	GACGGTATT	CGGATGCTG	IS inserted within the *adiA* gene
	IS1H	5066636..5067399	CCGGTAAAC	CTTCTGATG	IS inserted within an intergenic region
IS200/ IS605_ssgr_IS200	IS200C	1127230..1127936	**TTTT**	**TTTT**	Single IS transposition. IS inserted within a T-rich region
	IS200C	1690413..1691121	**TTTT**	**TTTT**	Single IS transposition. IS inserted within a T-rich region
	IS200C	2442570..2443280	**TTAA**	**TTAA**	Single IS transposition. IS inserted within a T-rich region
	IS200C	2481694..2482403	**TTTT**	**TTAT**	Single IS transposition. IS inserted within a T-rich region
	IS200C	2990220..2990930	**AAAA**	**AAAA**	Single IS transposition. IS inserted within a T-rich region
	IS200C	3058643..3059351	**TAAA**	**AAAA**	Single IS transposition. IS inserted within a T-rich region
	IS200C	3060222..3060929	**AAAA**	**AAAA**	Single IS transposition. IS inserted within a T-rich region
	IS200C	3271558..3272271	GCAA	AAAA	IS inserted within a putative prophage
	IS200C	3939865..3940573	**CAAA**	**AAAA**	Single IS transposition. IS inserted within a T-rich region
	IS200C	3994005..3994713	**AAAA**	**AAAA**	Single IS transposition. IS inserted within a T-rich region
IS3	IS600	3254256..3255501	CAA	ACA	IS inserted within a genomic island
	ISSd1	949559..950499	CAGTT	-	IS (truncated) inserted within a putative prophage
	ISSd1	3267154..3267978	-	GGT	IS (truncated) inserted within a genomic island
	ISSfl10	951719..952045	-	GTT	IS (truncated) inserted within a putative prophage
	IS3	3259199..3260456	TCAT	TTTA	IS inserted within a genomic island
	IS3	3236998..3237235	-	CTTC	IS (truncated) inserted within a genomic island
	ISEc52	3249338..3250086	-	-	IS (truncated) inserted within a genomic island
	ISEc52	3246586..3247067	-	-	IS (truncated) inserted within a genomic island
ISAs1	ISEc1	369367..369900	-	CCCT	IS (truncated, formerly Rhs-rearrangement hot-spots element)
	ISEc1	2456311..2456957	GATC	-	IS (truncated, formerly Rhs-rearrangement hot-spots element)
	ISEc1	3675287..3676199	TGTTGTAG	TCCTTGGC	IS (formerly Rhs-rearrangement hot-spots element)
	ISEc1	3815490..3816780	GATGTATA	CCTGCTCA	IS (formerly Rhs-rearrangement hot-spots element)
	ISEc1	4160599..4161889	TTCCTTCC	CACTTCAC	IS (formerly Rhs-rearrangement hot-spots element)
	ISEc1	5069737..5071026	AGACCAGT	GCATGTCA	IS (formerly Rhs-rearrangement hot-spots element)
IS6	IS26	4500893..4501712	**AAATCATG**	ATATCAAG	Composite transposon harboring the *bla*_TEM-1B_ (two copies), *catA1*, *aph(6′)-id*, *aph(3′’)-ib, sul2*, and *tetD* genes.
	IS26	4503629..4504448	ATATCGGC	GGTAAATC	
	IS26	4509192..4510011	CCGGCAAT	GTAAGCTG	
	IS26	4513665..4514484	ACCATTTG	CGCTGCGG	
	IS26	4515814..4516633	CAACAGGG	**AAATCATG**	
IS200/ IS605	IS609	3978710..3980457	CTCA	ATAA	IS inserted within the *yajI* gene
	IS609	4689442..4691189	TGTG	ATAA	IS inserted within an intergenic region
	IS609	2110716..2111379	-	-	IS (truncated) inserted within the *yedK* gene
	ISEc46	2191062..2192824	TCAT	CTAA	IS inserted within an intergenic region
IS3 ssgr IS150	IS1397	1214273..1215704	**TCAA**	**TCAA**	Single IS transposition within an intergenic region
	IS1397	1368490..1369921	**TGGC**	**TGGC**	Single IS transposition within an intergenic region
	IS150	259853..261295	**AAG**	**AAG**	Single IS transposition within an intergenic region
	IS150	2414087..2415529	**GTT**	**GTT**	Single IS transposition. IS inserted within a genomic island
IS3_ssgr_IS2	IS2	937126..938456	GTGGT	TTGTC	IS inserted within a putative prophage
	IS2	966497.. 967827	CCGCC	ACGGT	IS inserted within a putative prophage
	IS2	2027528..2028858	**CCTTT**	**CCTTT**	Single IS transposition. IS inserted within a genomic island
	IS2	4799912..4800262	AAAAC	-	IS (truncated) inserted within a putative prophage
IS21	IS100Kyp	2015511..2017464	**TTTGT**	**TTTGT**	Single IS transposition. IS inserted within a genomic island
	IS100Kyp	3273162..3275115	GTGATAAC	GATAACAT	IS inserted within a genomic island
	IS100Kyp	4582722.. 4584675	TTCAGATG	AGATGTAT	IS inserted within a putative prophage
IS66	IS682	924827..926816	-	CATGTATC	IS (truncated) inserted within a putative prophage
	ISEc22	923252..924827	ACAGAAGG	-	IS (truncated) inserted within a putative prophage
	ISCro1	946022.. 948720	**TTTTATCT**	**TTTTATCT**	Single IS transposition. IS inserted within a putative prophage
IS3_ssgr_IS51	IS629	570569..571878	**ATT**	**ATT**	IS inserted within the *acrF* gene
	IS1203	971759..973068	GATTACTG	GTAATATC	IS inserted within a putative prophage
ISL3	ISKox3	970324..971101	-	ATGTATCA	IS (truncated) inserted within a putative prophage
	ISEc38	2022594..2024315	AAAAGT	ACTTTT	Single IS transposition. IS inserted within a genomic island (inverted TSD)
IS481	ISErp1	891175.. 892368	**TATAATG**	**TATAATG**	Single IS transposition. IS inserted within a putative prophage
IS30	IS30D	950498..951718	**GT**	**GT**	Single IS transposition. IS inserted within a putative prophage
IS4	IS10A	105162..106490	GGCCGAGC	GTGCTGAAC	IS inserted into IS1-composite transposon
IS1380	ISEcp1	326913..330008	**TTTA**	**TTTA**	Single IS transposition. IS inserted within a hypothetical gene
IS110	IS5075	1568363..1569689	**TT**	**TT**	Single IS transposition. IS inserted within a hypothetical gene

## References

[1] Tacconelli E., Carrara E., Savoldi A., Harbarth S., Mendelson M., Monnet D.L., Pulcini C., Kahlmeter G., Kluytmans J., Carmeli Y. (2018). Discovery, research, and development of new antibiotics: The WHO priority list of antibiotic-resistant bacteria and tuberculosis. Lancet Infect. Dis..

[2] Oteo J., Hernandez J.M., Espasa M., Fleites A., Saez D., Bautista V., Perez-Vazquez M., Fernandez-Garcia M.D., Delgado-Iribarren A., Sanchez-Romero I. (2013). Emergence of OXA-48-producing *Klebsiella pneumoniae* and the novel carbapenemases OXA-244 and OXA-245 in Spain. J. Antimicrob. Chemother..

[3] Valenza G., Nickel S., Pfeifer Y., Eller C., Krupa E., Lehner-Reindl V., Holler C. (2014). Extended-spectrum-beta-lactamase-producing *Escherichia coli* as intestinal colonizers in the German community. Antimicrob. Agents Chemother..

[4] Potron A., Poirel L., Dortet L., Nordmann P. (2016). Characterisation of OXA-244, a chromosomally-encoded OXA-48-like beta-lactamase from *Escherichia coli*. Int. J. Antimicrob. Agents.

[5] Hoyos-Mallecot Y., Naas T., Bonnin R.A., Patino R., Glaser P., Fortineau N., Dortet L. (2017). OXA-244-Producing *Escherichia coli* Isolates, a Challenge for Clinical Microbiology Laboratories. Antimicrob. Agents Chemother..

[6] Findlay J., Hopkins K.L., Loy R., Doumith M., Meunier D., Hill R., Pike R., Mustafa N., Livermore D.M., Woodford N. (2017). OXA-48-like carbapenemases in the UK: An analysis of isolates and cases from 2007 to 2014. J. Antimicrob. Chemother..

[7] van Hattem J.M., Arcilla M.S., Bootsma M.C., van Genderen P.J., Goorhuis A., Grobusch M.P., Molhoek N., Oude Lashof A.M., Schultsz C., Stobberingh E.E. (2016). Prolonged carriage and potential onward transmission of carbapenemase-producing Enterobacteriaceae in Dutch travelers. Future Microbiol..

[8] Beyrouthy R., Robin F., Delmas J., Gibold L., Dalmasso G., Dabboussi F., Hamze M., Bonnet R. (2014). IS1R-mediated plasticity of IncL/M plasmids leads to the insertion of bla OXA-48 into the *Escherichia coli* Chromosome. Antimicrob. Agents Chemother..

[9] Wirth T., Falush D., Lan R., Colles F., Mensa P., Wieler L.H., Karch H., Reeves P.R., Maiden M.C., Ochman H. (2006). Sex and virulence in *Escherichia coli*: An evolutionary perspective. Mol. Microbiol..

[10] Brzuszkiewicz E., Bruggemann H., Liesegang H., Emmerth M., Olschlager T., Nagy G., Albermann K., Wagner C., Buchrieser C., Emody L. (2006). How to become a uropathogen: Comparative genomic analysis of extraintestinal pathogenic *Escherichia coli* strains. Proc. Natl. Acad. Sci. USA.

[11] Wang J., Li X., Li J., Hurley D., Bai X., Yu Z., Cao Y., Wall E., Fanning S., Bai L. (2017). Complete genetic analysis of a *Salmonella enterica* serovar Indiana isolate accompanying four plasmids carrying mcr-1, ESBL and other resistance genes in China. Vet. Microbiol..

[12] Yang Q.E., Walsh T.R. (2017). Toxin-antitoxin systems and their role in disseminating and maintaining antimicrobial resistance. FEMS Microbiol. Rev..

[13] Zhang C., Feng Y., Liu F., Jiang H., Qu Z., Lei M., Wang J., Zhang B., Hu Y., Ding J. (2017). A Phage-Like IncY Plasmid Carrying the mcr-1 Gene in *Escherichia coli* from a Pig Farm in China. Antimicrob. Agents Chemother..

[14] Siguier P., Gourbeyre E., Chandler M. (2014). Bacterial insertion sequences: Their genomic impact and diversity. FEMS Microbiol. Rev..

[15] He S., Hickman A.B., Varani A.M., Siguier P., Chandler M., Dekker J.P., Dyda F. (2015). Insertion Sequence IS26 Reorganizes Plasmids in Clinically Isolated Multidrug-Resistant Bacteria by Replicative Transposition. MBio.

[16] Marquez-Ortiz R.A., Haggerty L., Olarte N., Duarte C., Garza-Ramos U., Silva-Sanchez J., Castro B.E., Sim E.M., Beltran M., Moncada M.V. (2017). Genomic Epidemiology of NDM-1-Encoding Plasmids in Latin American Clinical Isolates Reveals Insights into the Evolution of Multidrug Resistance. Genome Biol. Evol..

[17] Li H., Durbin R. (2010). Fast and accurate long-read alignment with Burrows-Wheeler transform. Bioinformatics.

[18] Milne I., Stephen G., Bayer M., Cock P.J., Pritchard L., Cardle L., Shaw P.D., Marshall D. (2013). Using Tablet for visual exploration of second-generation sequencing data. Brief. Bioinform..

[19] Krumsiek J., Arnold R., Rattei T. (2007). Gepard: A rapid and sensitive tool for creating dotplots on genome scale. Bioinformatics.

[20] Seemann T. (2014). Prokka: Rapid prokaryotic genome annotation. Bioinformatics.

[21] Rutherford K., Parkhill J., Crook J., Horsnell T., Rice P., Rajandream M.A., Barrell B. (2000). Artemis: Sequence visualization and annotation. Bioinformatics.

[22] Zankari E., Hasman H., Cosentino S., Vestergaard M., Rasmussen S., Lund O., Aarestrup F.M., Larsen M.V. (2012). Identification of acquired antimicrobial resistance genes. J. Antimicrob. Chemother..

[23] McArthur A.G., Waglechner N., Nizam F., Yan A., Azad M.A., Baylay A.J., Bhullar K., Canova M.J., De Pascale G., Ejim L. (2013). The comprehensive antibiotic resistance database. Antimicrob. Agents Chemother..

[24] Gupta S.K., Padmanabhan B.R., Diene S.M., Lopez-Rojas R., Kempf M., Landraud L., Rolain J.M. (2014). ARG-ANNOT, a new bioinformatic tool to discover antibiotic resistance genes in bacterial genomes. Antimicrob. Agents Chemother..

